# Phenotyping Type 2 Diabetes in Terms of Myocardial Insulin Resistance and Its Potential Cardiovascular Consequences: A New Strategy Based on ^18^F-FDG PET/CT

**DOI:** 10.3390/jpm12010030

**Published:** 2022-01-02

**Authors:** José Raul Herance, Rafael Simó, Mayra Alejandra Velasquez, Bruno Paun, Daniel García-Leon, Carolina Aparicio, Roso Marés, Olga Simó-Servat, Joan Castell-Conesa, Cristina Hernández, Santiago Aguadé-Bruix

**Affiliations:** 1 Medical Molecular Imaging Research Group, Nuclear Medicine Department, Vall d’Hebron Research Institute (VHIR), Vall d’Hebron University Hospital, Autonomous University Barcelona, 08035 Barcelona, Spain; brunopaun@gmail.com (B.P.); daniel.garcia.leon@ki.se (D.G.-L.); carolina.aparicio@vhir.org (C.A.); rousymares@gmail.com (R.M.); jcastell@simm.barcelona (J.C.-C.); saguade@vhebron.net (S.A.-B.); 2 CIBERBBN, Instituto de Salud Carlos III, 28029 Madrid, Spain; 3 Diabetes and Metabolism Research Group, VHIR, Endocrinology Department, Vall d’Hebron University Hospital, Autonomous University Barcelona, 08035 Barcelona, Spain; mvelasquez@vhebron.net (M.A.V.); olga.simo@vhir.org (O.S.-S.); cristina.hernandez@vhir.org (C.H.); 4 CIBERDEM, Instituto de Salud Carlos III, 28029 Madrid, Spain

**Keywords:** myocardial insulin resistance, type 2 diabetes, cardiovascular risk, ^18^F-FDG PET/CT

## Abstract

Background: Systemic insulin resistance is generally postulated as an independent risk factor of cardiovascular events in type 2 diabetes (T2D). However, the role of myocardial insulin resistance (mIR) remains to be clarified. Methods: Two ^18^F-FDG PET/CT scans were performed on forty-three T2D patients at baseline and after hyperinsulinemic–euglycemic clamp (HEC). Myocardial insulin sensitivity (mIS) was determined by measuring the increment in myocardial ^18^F-FDG uptake after HEC. Coronary artery calcium scoring (CACs) and myocardial radiodensity (mRD) were assessed by CT. Results: After HEC, seventeen patients exhibited a strikingly enhancement of myocardial ^18^F-FDG uptake and twenty-six a marginal increase, thus revealing mIS and mIR, respectively. Patients with mIR showed higher mRD (HU: 38.95 [33.81–44.06] vs. 30.82 [21.48–38.02]; *p* = 0.03) and CACs > 400 (AU: 52% vs. 29%; *p* = 0.002) than patients with mIS. In addition, HOMA-IR and mIS only showed a correlation in those patients with mIR. Conclusions: ^18^F-FDG PET combined with HEC is a reliable method for identifying patients with mIR. This subgroup of patients was found to be specifically at high risk of developing cardiovascular events and showed myocardial structural changes. Moreover, the gold-standard HOMA-IR index was only associated with mIR in this subgroup of patients. Our results open up a new avenue for stratifying patients with cardiovascular risk in T2D.

## 1. Introduction

Type 2 diabetes mellitus (T2D) is a metabolic disorder that accounts for approximately 90% of all diabetic patients [1,2]. Currently, there are more than 400 million T2D patients worldwide and its prevalence is increasing annually [3,4]. This disorder causes more than 3 million deaths per year, mainly by cardiovascular disorders, and is expected to double in cases and become the seventh cause of death worldwide by 2030 [5,6]. Thus, it is crucial to increase our knowledge about T2DM pathophysiology in order to reduce its alarming annual incidence rate and improve the management of its associated comorbidities. T2D is a chronic inflammatory disease characterized by elevated plasma glucose levels arising from pancreatic β-cell dysfunction and inefficient action of insulin in cells (insulin resistance (IR)) in insulin-sensitive (IS) organs and tissues such as the skeletal muscle, liver or heart [4,7].

Many clinical trials have shown that systemic insulin resistance is an independent risk factor for heart failure and cardiovascular death [1]. In addition, increasing evidence points to insulin resistance as the primary etiologic factor in the development of nonischemic heart failure [2,3]. Furthermore, a number of preclinical studies have additionally shown that myocardial insulin resistance (mIR), which is characterized by inefficient energy metabolism, co-occurs with systemic insulin resistance and contributes to post-ischemic heart failure [4]. However, a method to delineate mIR in patients with T2D is still an unmet clinical need.

Positron emission tomography/computed tomography (PET/CT) is a molecular medical imaging technique used for clinical diagnosis to observe the expression of biological targets or physiological processes in vivo [5,6]. PET provides mainly functional information of the body, whereas CT gives us the anatomical characteristics. The PET image is obtainable thanks to the use of radioactive probes known as radiopharmaceuticals (RFs), which define the biological objectives to be evaluated. Among all RFs, fluorine-18 fluorodeoxyglucose (^18^F-FDG) is the most widely used and available in nuclear medicine departments [8]. This RF allows the observation of the glucose metabolization process and is used for different objectives in different medical areas such as oncology, neurology, cardiology, endocrinology or infectious diseases either in clinical examinations or in clinical trials [8].

Here, we conducted a proof of concept using ^18^F-FDG PET/CT performed at baseline (fasting state) and after hyperinsulinemic–euglycemic clamp (HEC) as a method to determine myocardial insulin sensitivity (mIS) in each patient in order to gain new insights into mIR and its clinical consequences.

## 2. Materials and Methods

### 2.1. Subjects

This pilot clinical trial comprised forty-seven patients with T2D prospectively recruited from February 2018 to July 2019 at the Outpatient’s Department of Endocrinology of Vall d’Hebron University Hospital. The clinical characteristics of the forty-three patients included in the study are shown in Table 1.

The inclusion criteria were: (1) a clinical diagnosis of T2D within the last 5 years, (2) aged between 50 and 79 years. The exclusion criteria were: (1) a clinical diagnosis of type 1 diabetes and/or (2) any type of cardiovascular disease (CVD), (3) any absolute or relative contraindication to PET/CT (e.g., claustrophobia), (4) any concomitant disease associated with a short life expectancy.

### 2.2. Characterization of Myocardial Insulin Resistance (mIR)

Two ^18^F-FDG PET/CT scans were performed for each patient in a random order within 2 days under at least 8 h of fasting conditions and after the withdrawal of any medication the day before. A dose of 1.9 MBq/Kg of ^18^F-FDG was iv administered to patients before each scan session. A whole-body baseline PET/CT scan was performed 60 min after ^8^F-FDG injection for 12 min, followed by a 6 min of cardiac PET scan (baseline scan). A similar PET/CT scanning session was conducted after HEC (HEC scan). The HEC procedure was performed as previously reported with minor modifications [9,10]. Before the HEC procedure, a blood sample was withdrawn for biochemical analysis at the Biochemistry Core Facilities of Vall d’Hebron University Hospital using standardized and validated routine methodologies. For the HEC scan, ^18^F-FDG was administered at least 1.5 h after starting the HEC procedure and only when three consecutive plasma glucose values of 100 ± 15 mg/dl separated by 5 min intervals were obtained. HEC was maintained for 60 min after ^18^F-FDG injection as previously described [11,12]. Then, HEC was stopped and patients were scanned, using the same protocol used for the baseline scan. Myocardial insulin sensitivity (mIS), understood as the inverse of mIR, was determined as the difference in standardized uptake value (ΔSUV) between the HEC and baseline scans. Using patients as their own controls allowed us to accurately identify those patients with mIR.

### 2.3. PET/CT Acquisition

All acquired ^18^F-FDG PET images in DICOM format were first SUVbw normalized using the PET DICOM Extension available in 3D Slicer [13,14]. PET/CT imaging was acquired using a Biograph mCT 64S scanner (Siemens Healthcare, Erlangen, Germany). Coronary synchronized CT calcium score acquisition was performed after the PET scan with the following acquisition parameters: tube voltage = 80 kV, pitch = 0.9 pixel, spacing = 0.7168 mm isotropic, tube current = 126 mA, exposure time = 0.5 s, image matrix size = 512² and slice thickness = 0.6 mm.

Imaging data were reconstructed with 3 iterations and 21 subsets and Gaussian filtering (order 3), with attenuation, scatter and point spread function corrections, and also the application of time of flight methods. Pixel spacing was 2.03642 × 2.03642 mm, matrix size was 200² with a slice thickness of 3 mm for the whole body, and for the cardiac bed we used Gaussian filtering (order 3) with 3 iterations and 21 subsets, all corrections, ZOOM value 2. Pixel spacing was 1.59095 × 1.59095 mm and matrix size was of 256² with a slice thickness of 2.027 mm.

### 2.4. Data Processing and Analysis

After normalizing PET data by body weight and injected dose, PET images and CT images were cropped to show only the heart and neighboring ascending and descending aorta and saved in NIfTI format. Image analysis was performed with Carimas software (version 2.9, Turku, Finland Proper, Finland). The myocardium of the normalized HEC scan image at the first time point was automatically segmented using Carimas’s automatic segmentation tool, followed with manual adjustment improvement when necessary, while taking into consideration anatomical information from the co-registered CT image. Cardiac segmentations were evaluated and approved by nuclear medicine and radiologists specialized in cardiac imaging. Once the myocardium was segmented, total SUVbw values were obtained. Calcium scores were determined using a semi-automatic methodology using syngo.via cardiac CT software (version 5.01, Siemens Healthineers, Erlangen, Germany). Patients were classified based on their Agatston units (AU) as low–moderate risk (<400 AU) or high risk (>400 AU).

### 2.5. Statistical Analysis

A Kolmogorov–Smirnov test was used for group comparisons and Spearman’s correlation analysis for variable associations. *p* values below 0.05 were considered statistically significant. All data were analyzed in GraphPad Prism (Version 6.01, San Diego, CA, USA).

## 3. Results

Four patients dropped out of the study before their PET/CT assessments were completed and were, therefore, excluded from the analysis of the results. The clinical characteristics of the remaining forty-three patients included in the study are shown in Table 1.

**Figure 1:**
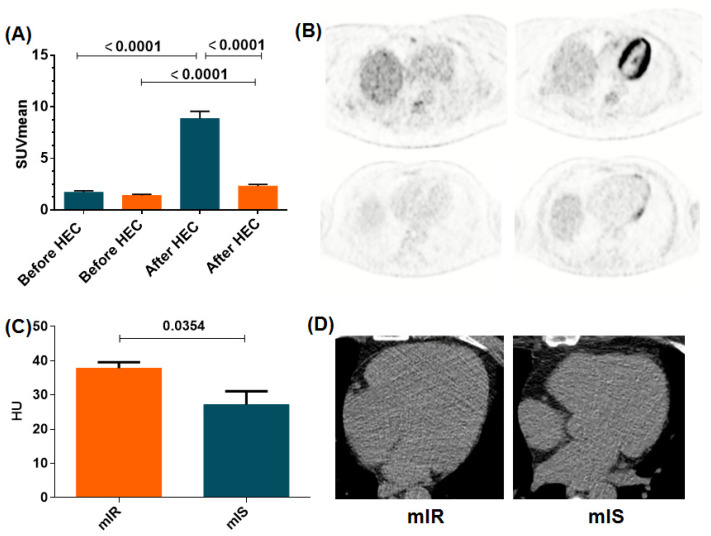
(**A**) LV myocardial ^18^F-FDG uptake before and after hyperinsulinemic–euglycemic clamp (HEC) conditions in both phenotypes of T2D patients (mIR and mIS in orange and blue, respectively). (**B**) Representative PET/CT images showing myocardial ^18^F-FDG uptake in two patients from the mIS (top) and mIR group (bottom), respectively, in baseline scan (left) and HEC scan (right) conditions. (**C**) Values of mRD (HU) in the mIR and mIS groups (38.95 [33.81–44.06] vs. 30.82 [21.48–38.02]; *p* = 0.03). (**D**) Representative cardiac CT images from patients exhibiting mIR (left) or mIS (right). Results are displayed as median ± SEM.

All patients showed low left ventricle myocardial ^18^F-FDG uptake at the baseline scan, close to the background-to-noise signal. However, after HEC, 17 (39.5%) showed a pronounced enhancement in myocardial ^18^F-FDG uptake (1.59 [1.42–1.92] SUV vs. 7.67 [7.10–9.49] SUV) indicative of an insulin-sensitive myocardium (mIS), whereas 26 (60.4%) showed only a marginal increase in ^18^F-FDG uptake (1.18 [1.06–1.59] SUV vs. 2.27 [1.62–2.93] SUV), thus revealing the presence of mIR (Figure 1A,B).

**Figure 2:**
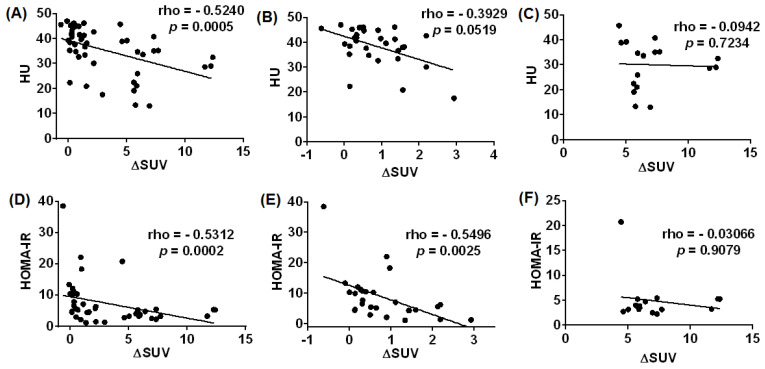
Correlation analysis between ΔSUV and: (1) mRD (HU) in all T2D patients (**A**), patients with mIR (**B**) and mIS (**C**) and (2) HOMA-IR in all T2D patients (**D**) and patients with mIR (**E**) and mIS (**F**).

Myocardial radiodensity (mRD) was significantly higher in the mIR group than in patients showing mIS (Figure 1C,D) (38.95 [33.81–44.06] AU vs. 30.82 [21.48–38.02] AU; *p* = 0.03) (Table 2). In addition, a negative correlation was found between mRD and ΔSUV using all patients (rho = −0.5238; *p* = 0.0005) and a clear trend for the mIR group (rho = −0.3924; *p* = 0.0519) but not the mIS group (rho = −0.0942; *p* = 0.7234) (Figure 2A–C).

Regarding the relationship between mIR and cardiovascular risk factors, we found that patients with mIR did not present significantly higher levels of HbA1c, cholesterol or blood pressure but exhibited significantly higher HOMA-IR (5.51 [4.39–9.98] vs. 3.88 [3.21–5.26]; *p* = 0.03) and CACs (52% vs. 29%; *p* = 0.002) (Table 2).

Finally, a negative correlation between HOMA-IR and ΔSUV was observed in those patients with mIR (rho = −0.5496; *p* = 0.0025), but not in those with mIS (Figure 2E,F).

## 4. Discussion

This pilot study revealed that after HEC two phenotypes in terms of insulin-mediated myocardial ^18^F-FDG uptake can be distinguished, insulin sensitive (mIS) and insulin resistant (mIR). This finding suggests that in the fasting stage a low uptake of glucose exists. However, in the post-prandial state two different patterns of myocardial metabolism can be detected: (1) a physiological response: high uptake of glucose mediated by insulin, mIS; (2) a pathological response: a low glucose uptake due to mIR. Since under HEC conditions the levels of circulating insulin were similar to those reported in the post-prandial state it can be postulated that in physiological conditions a cyclic glucose uptake in relation to the post-prandial state occurs, but this is not the case for those patients with mIR in whom the uptake of glucose is low at all times. In this regard, it is well established that free fatty acids (FFAs) represent the primary fuel for the myocardium in the fasting state while glucose does so in the fed state [15], but it seems that the latter is impaired in the mIR group of T2D patients. The clinical significance of these findings in terms of myocardial function remains to be elucidated.

Moreover, we have found that the presence of mIR is frequent (around 60%) in patients with T2D without established cardiovascular disease. Notably, in these patients we found a relationship between patients with mIR and systemic HOMA-IR. These finding shed light onto the inconsistencies often found in the relationship between the systemic insulin resistance index and cardiovascular events in T2D.

Patients with mIR presented higher mRD than those exhibiting mIS. Additionally, a significant negative correlation between myocardial ^18^F-FDG uptake and radiodensity was observed. These findings suggest that the metabolic abnormalities induced by mIR not only lead to functional, but also to structural, changes in the myocardium. Furthermore, a clear relationship between mIR and CACs was detected. Thus, patients with mIR presented a higher frequency of CACs > 400 AU than patients with mIS. Since CACs is the most sensitive cardiovascular risk stratification tool among asymptomatic patients with diabetes [16,17], our results suggest that patients with mIR are at very high risk of developing cardiovascular events.

There are some limitations in this study such as the lack of comparison with a healthy control group, and the reduced number of patients with T2D who were enrolled in the clinical trial. The main reason is because PET/CT combined with HEC is cumbersome and time-consuming and it has to be performed twice in the same patient. In addition, PET/CT is a radioactive technique. Therefore, more accessible and safe biomarkers or surrogates of mIR are needed. Furthermore, studies on the long-term clinical consequences of mIR, and in particular whether this may be a significant underlying mechanism of diabetic cardiomyopathy, are needed.

## 5. Conclusions

^18^F-FDG PET combined with HEC is a reliable method that allows a clear identification of patients with mIR. This subset of T2D patients shows structural changes in the myocardium and they have a high proportion of CACs > 400 AU. Further prospective research is required, not only to confirm these findings but also to determine the global impact of mIR on myocardium remodeling, functionality and cardiovascular outcomes.

## Figures and Tables

**Table 1:** Clinical characteristics of patients included in the study.

	N = 47
Age (years)	67 ± 7
Gender (M/F)	24/23
BMI (Kg/m^2^)	30.76 [28.74–35.11]
Waist (cm)	108 [99–117]
HOMA-IR	4.76 [3.22–6.58]
HbA1c (%)	7.30 [6.60–7.50]
HbA1c (mmol/mol)	56 [51–58]
Total Cholesterol (mmol/L)	4.24 [3.83–5.07]
HDL-C (mmol/L)	1.19 [1.01–1.35]
LDL-C (mmol/L)	2.38 [2.02–2.97]
Triglycerides (mmol/L)	1.26 [0.94–1.99]
CACs > 400 AU (% patients)	42.5
mRD (HU)	36.05 [28.99–41.16]
ΔSUV	1.56 [0.55–5.82]

Data are expressed as mean ± SD or median [interquartile range]. CACs: coronary artery calcium score; mRD: myocardial radiodensity; SUV: standardized uptake value.

**Table 2:** Anthropometrical, biochemical and myocardial features of patients with mIS and mIR.

	mIS (N = 21)	mIR (N = 26)	*p*
Age (years)	70 ± 8	66 ± 6	0.18
Gender (M/F)	11/10	13/13	0.99
BMI (Kg/m^2^)	30.46 [27.83–37.22]	31.16 [29.27–34.87]	0.50
Waist (cm)	107 [99–117]	109 [100–115]	0.82
HOMA-IR	3.88 [3.21–5.26]	5.51 [4.39–9.98]	0.03
Hb1Ac (%)	7.05 [6.48–7.40]	7.40 [6.80–7.70]	0.35
HbA1c (mmol/mol)	54 [47–57]	57 [51–61]	0.35
Total Cholesterol (mmol/L)	4.17 [3.65–4.43]	4.56 [4.01–5.36]	0.17
HDL-C (mmol/L)	1.24 [0.98–1.34]	1.11 [0.98–1.29]	0.30
LDL-C (mmol/L)	2.17 [1.97–2.64]	2.72 [2.12–3.16]	0.30
Triglycerides (mmol/L)	1.23 [0.82–1.87]	1.4 [1.29–2.19]	0.25
CACs > 400 AU (% patients)	29	52	<0.01
mRD (HU)	30.82 [21.48–38.02]	38.95 [33.81–44.06]	<0.01
ΔSUV	6.165 [5.625–7.615]	0.7750 [0.2675–1.468]	<0.0001

Data are expressed as mean ± SD or median [interquartile range]. CACs: coronary artery calcium score; mRD: myocardial radiodensity; SUV: standardized uptake value.

## Data Availability

All data used for the current study are available from the corresponding authors on reasonable request.
